# Notochord-derived hedgehog is essential for tail regeneration in *Xenopus* tadpole

**DOI:** 10.1186/1471-213X-14-27

**Published:** 2014-06-18

**Authors:** Yuka Taniguchi, Kenji Watanabe, Makoto Mochii

**Affiliations:** 1 Department of Life Science, Graduate school of Life Science, University of Hyogo, 3-2-1 Koto, Kamigori Akou, Hyogo 678-1297, Japan; 2 Present address: Technische Universität Dresden, DFG-Center for Regenerative Therapies Dresden, Fetscherstrasse 105, Dresden 01307, Germany

**Keywords:** *Xenopus*, Tail regeneration, Hedgehog, Cyclopamine, Notochord, Spinal cord

## Abstract

**Background:**

Appendage regeneration in amphibians is regulated by the combinatorial actions of signaling molecules. The requirement of molecules secreted from specific tissues is reflected by the observation that the whole process of regeneration can be inhibited if a certain tissue is removed from the amputated stump. Interestingly, urodeles and anurans show different tissue dependencies during tail regeneration. The spinal cord is essential for tail regeneration in urodele but not in anuran larva, whereas the notochord but not the spinal cord is essential for tail regeneration in anuran tadpoles. Sonic hedgehog is one of the signaling molecules responsible for such phenomenon in axolotl, as hedgehog signaling is essential for overall tail regeneration and *sonic hedgehog* is exclusively expressed in the spinal cord. In order to know whether hedgehog signaling is involved in the molecular mechanism underlying the inconsistent tissue dependency for tail regeneration between anurans and urodeles, we investigated expression of hedgehog signal-related genes in the regenerating tail of *Xenopus* tadpole and examined the effect of the hedgehog signal inhibitor, cyclopamine, on the tail regeneration.

**Results:**

In *Xenopus*, sonic hedgehog is expressed exclusively in the notochord but not in the spinal cord of the regenerate. Overall regeneration was severely impaired in cyclopamine-treated tadpoles. Notochord maturation in the regenerate, including cell alignment and vacuolation, and myofiber formation were inhibited. Proliferation of spinal cord cells in the neural ampulla and of mesenchymal cells was also impaired.

**Conclusion:**

As in the axolotl, hedgehog signaling is required for multiple steps in tail regeneration in the *Xenopus* tadpole, although the location of the Shh source is quite different between the two species. This difference in Shh localization is the likely basis for the differing tissue requirement for tail regeneration between urodeles and anurans.

## Background

Regeneration of amphibian appendages, including the tail, represents a valuable model system to analyze molecular mechanisms underlying cell growth, cell differentiation, morphogenesis and pattern formation in epimorphic regeneration (reviewed in [1-5]). Multiple signaling molecules have critical roles both in anuran and urodele tail regeneration. For example, larval tail regeneration in *Xenopus* is regulated by bone morphogenetic protein (BMP), Notch, fibroblast growth factors (FGFs), transforming growth factor (TGF)–β, canonical Wnt and non-canonical Wnt [6-12]. Hedgehog signaling is required for axolotl tail regeneration [13]. A significant role of FGFs and Wnts has been suggested by their expression patterns during tail regeneration in adult or larval salamander [14-17]. Therefore, a considerable number of signaling mechanisms are thought to be shared between anurans and urodeles during tail regeneration.

The requirement for signaling molecules in regeneration should be related, at least in part, to the phenomenon of the tissue dependency in appendage regeneration, which has been shown by ablating or destroying a specific tissue in the amputated stump [17-20]. The tissue dependency in tail regeneration is quite different between anurans and urodeles, although both species expressed overlapping set of genes for signaling molecules during tail regeneration. Urodele tail regeneration is completely dependent on the presence of the spinal cord [21], which is now known to be the source of FGFs and sonic hedgehog (Shh), while an ablation of the spinal cord results in a normal or partially defective regenerating tail in anuran larvae [12,18,19]. As hedgehog signalling is essential for overall tail regeneration in urodele [13], the exclusive expression of *shh* in the spinal cord is likely to be one of major reasons for the requirement of the spinal cord in urodele tail regeneration. It is interesting to know whether hedgehog signal is essential for the tail regeneration in anuran larvae, especially since the presence of the spinal cord is not required for anuran tail regeneration.

In this work we showed that tail regeneration in *Xenopus* larva is dependent on hedgehog signaling as in axolotl and that *shh* is expressed in the notochord but not in the spinal cord in the regenerating tadpole tail. Our results suggest that the different tissue specificity of *shh* expression is the major cause leading to the differences in the tissue dependency between anurans and urodeles.

## Results

### Expression of genes related to hedgehog signal during tail regeneration

**Figure 1:**
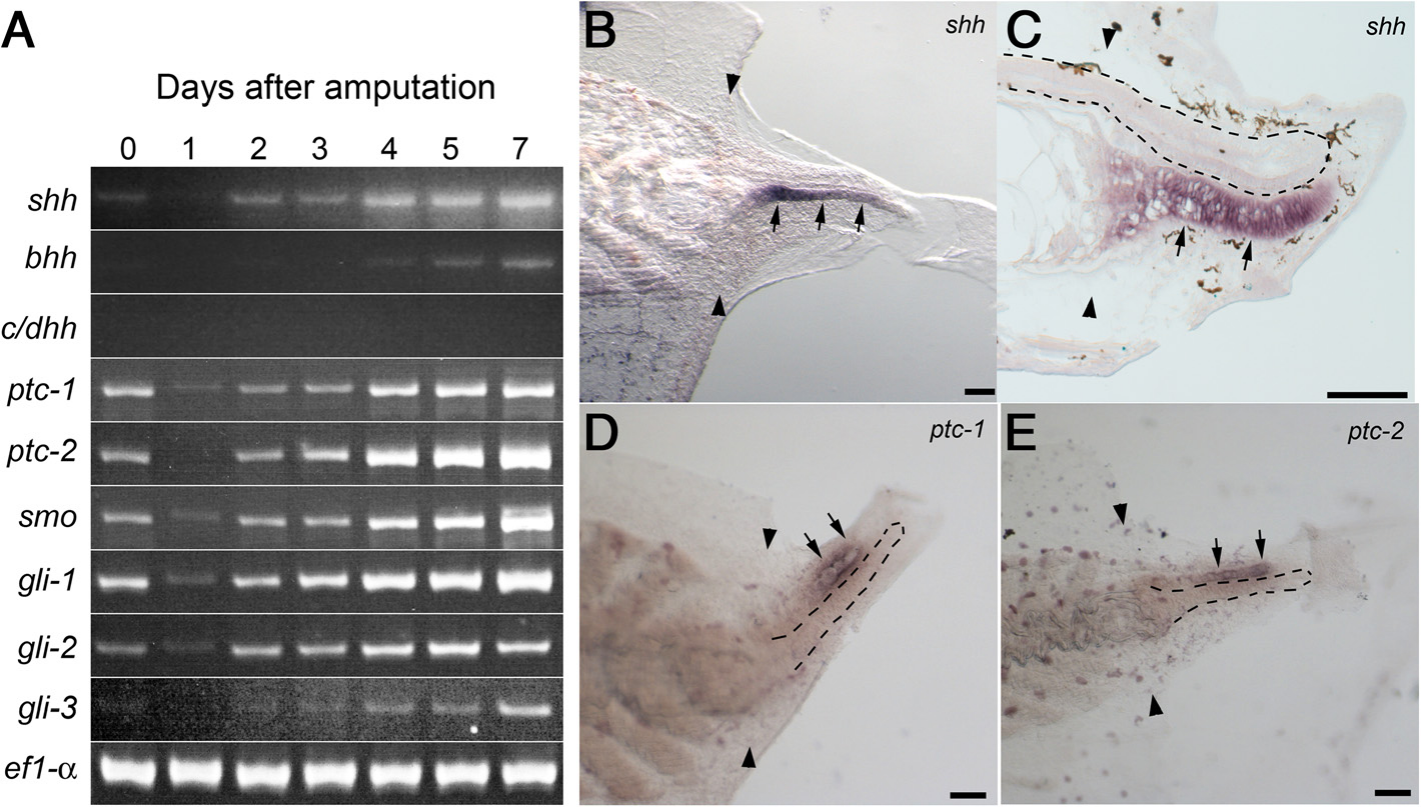
**Expression of hedgehog signal-related genes in regenerating tail. (A)** Semi-quantitative gene expression analysis by reverse transcription–polymerase chain reaction (RT–PCR) was carried out. RNA was isolated from amputated tails at the indicated day. Specific primer pairs and PCR conditions are indicated in Additional file 2: Table S1. **(B-E)** Whole mount **(B, D, E)** and section **(C)** in situ hybridization of regenerated tail. The spatial expression of *shh***(B, C)***ptc-1***(D)** and *ptc-2***(E)** was analyzed on the regenerating tail at day 4 **(C-E)** or day 5 **(B)**. Arrows indicate hybridization signal in the regenerating notochord **(B, C)** or spinal cord **(D, E)**. A pair of arrowheads marks the amputation plane. Black dashed lines indicate the shapes of the regenerating spinal cord **(C)** or notochord **(D, E)**. Bars, 100 μm.

*Xenopus laevis* have four hedgehog genes, *sonic hedgehog* (*shh)*, *banded hedgehog (bhh),* and the closely related *cephalic* and *desert hedgehogs*[22]. RT-PCR showed that *shh* and *bhh* were expressed in the regenerating tail but *cephalic/desert hedgehogs* were not (Figure 1A). In axolotl *shh* is exclusively expressed in the regenerating spinal cord during tail regeneration [13], whereas a previous study suggested that it is expressed in the regenerating notochord but not in the regenerating spinal cord in *Xenopus* larva [23]. In situ hybridization of whole mount samples and sagittal sections confirmed that *shh* signal was detected exclusively in the notochord in the entire regenerating region (Figure 1B, C). Expression signal for *bhh* was not detected in any tissue by in situ hybridization probably due to a low expression level (data not shown). These results show that the hedgehog ligands are produced exclusively or dominantly by the notochord in the regenerating tail in *Xenopus* in contrast to its exclusive expression in the spinal cord in axolotl tail.

RT-PCR showed that two *patched* genes (*ptc-1* and *ptc-2*), *smoothened* (*smo*) and three *gli* genes (*gli-1*, *gli-2* and *gli-3*) were also expressed in the regenerating tail (Figure 1A). Whole mount in situ hybridization revealed that *ptc-1* and *ptc-2* were expressed in the regenerating spinal cord (Figure 1D, E). Hybridization signal in other tissues, for example, notochord and muscle, was indistinguishable from the background staining. Expression of *gli* genes was not detected in any tissues by in situ hybridization (data not shown). These results support a possibility that hedgehog signal regulates growth and/or differentiation of regenerating tissues, including the spinal cord in *Xenopus*.

### Cyclopamine treatment suppresses tail regeneration in *Xenopus*

**Figure 2:**
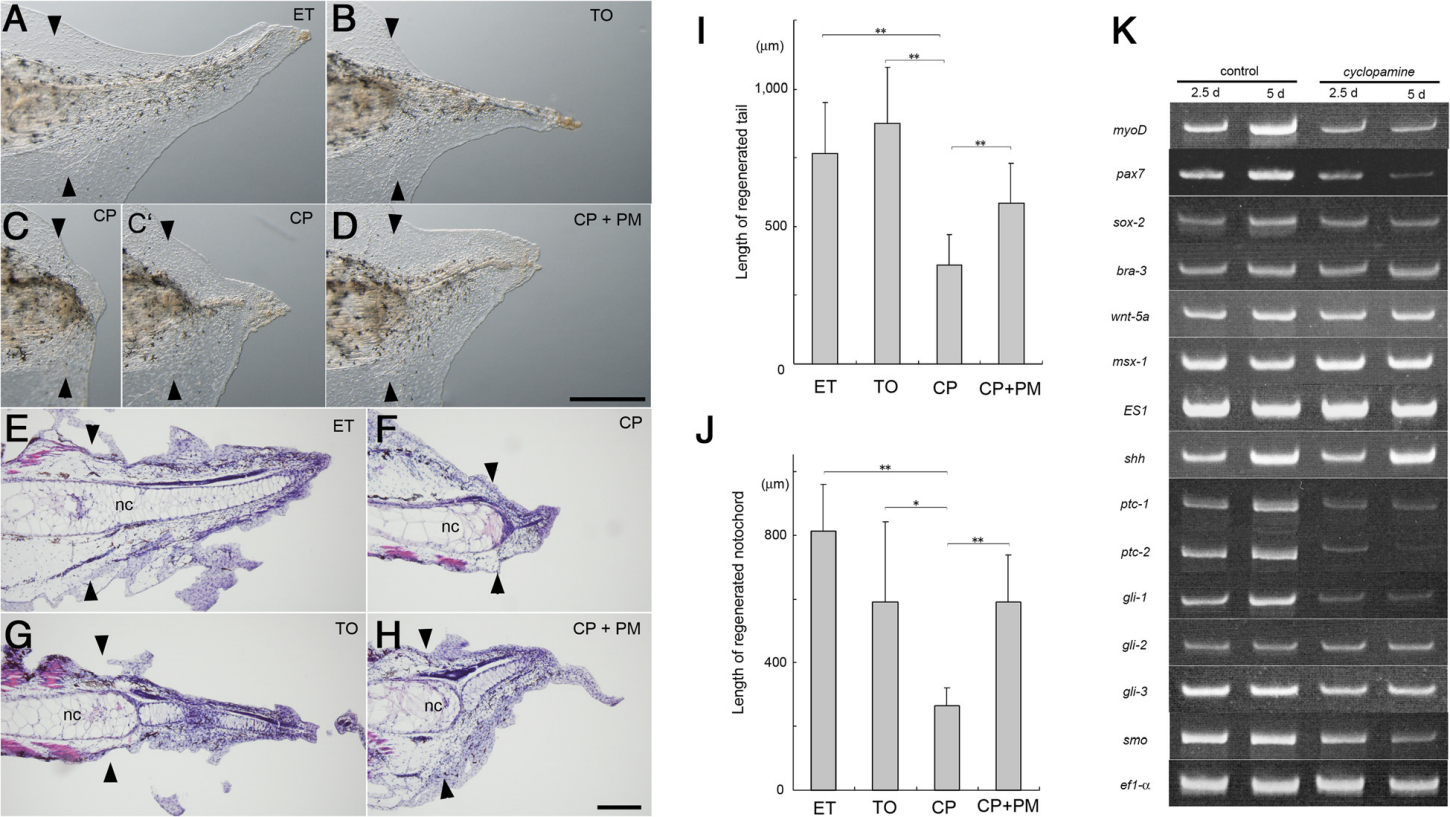
**Effect of cyclopamine on the tail regeneration.** The tail-amputated tadpole was maintained in the presence of the indicated compound. ET, 0.1% ethanol. TO, 2.5 μM tomatidine. CP, 2.5 μM cyclopamine. CP + PM, 2.5 μM cyclopamine and 0.25 μM purmorphamine. **(A-D)** Regenerated tails were observed under a stereoscopic microscope at day 7. Bar, 500 μm. **(E-H)** Histological analysis of the regenerated tail at day 7. Sagittal sections were stained with hematoxylin and eosin. nc, notochord. Bar, 100 μm. A pair of arrowheads marks the amputation plane. **(I, J)** The length of the regenerated tail and notochord at day 5. Mean length of the regenerated tail **(I)** or the notochord **(J)** was indicated with the standard deviation. Detail is shown in Tables 1 and 2. *, p-value < 0.05. **, p-value <0.01. **(K)** Gene expression analysis with RT-PCR. RNA from the control or cyclopamine-treated tails was analyzed at day 2.5 or day 5.

The DV patterning, blastema cell proliferation and cartilage differentiation in the regenerating axolotl tail are severely affected by cyclopamine, a widely used specific inhibitor for hedgehog signaling [13]. To test roles of hedgehog signal in *Xenopus* tail regeneration, the tail-amputated tadpoles were treated with cyclopamine. Growth of the regenerating tail was significantly retarded by cyclopamine supplied in the breeding water but was not affected by the same dosage of tomatidine, a control compound for cyclopamine (Figure 2A-C, I, Table 1). Similarly the length of the regenerating notochord was reduced by the cyclopamine treatment (Figure 2E-G, J, Table 2). Administration of pumorphamine, an agonist for hedgehog pathway, in the breeding water suppressed the effect of cyclopamine resulting in the nearly normal tail regeneration (Figure 2D, H-J), showing that the defects observed in the cyclopamine-treated tail were caused by the specific inhibition of the hedgehog signal.

**Table 1:** Effect of cyclopamine on length of regenerating tail

	**Day 3**				**Day 5**			
**Chemicals**	**ET**	**TO**	**CP**	**CP + PM**	**ET**	**TO**	**CP**	**CP + PM**
Number	11	11	12	11	9	9	12	10
Mean (μm)	725.3	785.2	401.2	487	1528.8	1751.6	718.4	1168.2
SD	209.3	225.7	171.9	134.1	375.2	408.1	219	293.1
P-value (ET)	-	0.26	0.2 E-4	2.4 E-3	-	0.12	2.7 E-6	1.6 E-2
P-value (TO)	-	-	7.5 E-5	6.1 E-4	-	-	2.2 E-7	1.1 E-2
P-value (CP)	-	-	-	0.1	-	-	-	2.7 E-4

Tail-amputated st. 48 tadpoles were maintained in water containing ethanol (ET, 0.1%), tomatidine (TO, 2.5 μM) or cyclopamine (CP, 2.5 μM) or cyclopamine plus purmorphamine (PM, 0.25 μM). Mean length of the regenerated tails was determined at day 3 and day 5. Student’s *t-*test was used to identify differences between indicated groups.

**Table 2:** Effect of cyclopamine on length of regenerating notochord

	**Day 3**				**Day 5**			
**Chemicals**	**ET**	**TO**	**CP**	**CP + PM**	**ET**	**TO**	**CP**	**CP + PM**
Number	8	8	8	8	8	8	7	8
Mean (μm)	301.5	274.5	198.5	254.9	811.3	590.7	263.3	591.9
SD	81.8	78.4	28.6	59.9	147.5	251.4	56.4	146.4
P-value (ET)	-	0.26	2.3 E-3	0.12	-	2.5 E-2	2.3 E-7	4.9 E-3
P-value (TO)	-	-	1.1 E-2	0.3	-	-	2.6 E-3	0.5
P-value (CP)	-	-	-	0.98	-	-	-	4.6 E-5

Tail-amputated st. 48 tadpoles were maintained in water containing ethanol (ET, 0.1%), tomatidine (TO, 2.5 μM), cyclopamine (CP, 2.5 μM), or cyclopamine plus purmorphamine (PM, 0.25 μM). Mean length of the regenerated notochord was determined at day 3 and day 5. Student’s *t-*test was used to identify differences between indicated groups.

TUNEL staining showed apoptotic cells in the distal region of the amputated tail at day 1 to day 3 in both cyclopamine-treated and control tadpoles (Additional file 1: Figure S1). The signals were found mainly in epidermis but rarely in notochord, spinal cord and mesenchymal region. It appeared that cyclopamine do not cause a significant change of the apoptotic cell number in this condition.

Gene expression in the regenerating tail was analyzed by RT-PCR (Figure 2K). Cyclopamine treatment resulted in down-regulation of several component genes for hedgehog signaling cascade, including *ptc-1*, *ptc-2*, *gli-1* and *smo*, indicating that these genes are targets of hedgehog signaling, although the expression level of *shh* was not affected. The down-regulation of *ptc-1* is consistent with the reduced expression of *ptc-1* in cyclopamine-treated axolotl tails [13]. The cyclopamine treatment down-regulated expression of *myoD* representing muscle tissue and slightly affected *sox-2* representing nerve tissues, although it did not affect *bra-3* representing the notochord or genes expressed in a broad range of regenerating tissues, such as *msx-1* and *wnt-5a*. Formation of normal wound epidermis was observed morphologically and confirmed by the unaffected expression of *ES-1*, a marker for the wound epidermis [11] (Figure 2K).

### Cyclopamine inhibits notochord differentiation

**Figure 3:**
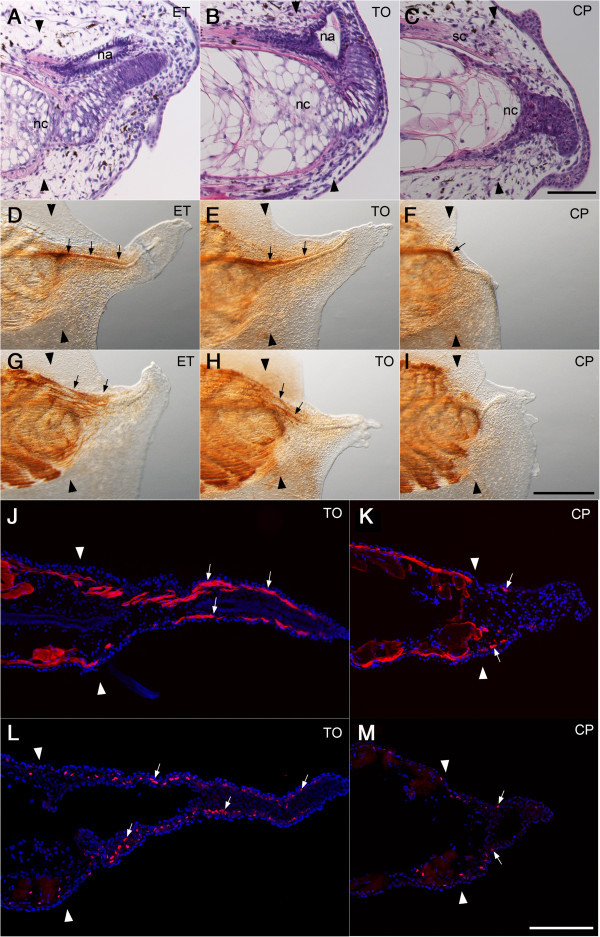
**Cyclopamine affects cell differentiation in the regenerating tail. (A-C)** Histological analysis of the regenerating tail at day 3. Sagittal sections were stained with hematoxylin and eosin. Cyclopamine suppressed the elongation and the vacuolation of the regenerating notochord cells. It also suppressed the formation of the neural ampulla **(C)**. nc, notochord. na, neural ampulla. sc, spinal cord. Bar, 200 μm. **(D-I)** Immunohistochemical detection of nerve cells and myofibers in the regenerating tail at day 5. Whole mount sample was immunostained with an anti-NCAM monoclonal antibody (4d) and a monoclonal antibody 12/101 to detect the nerve cells **(D-F)** and the myofibers **(G-I)**, respectively. Arrows indicate the regenerated spinal cord **(D-F)** and the regenerated myofibers **(G, H)**. Bar, 500 μm. **(J-K)** Immunofluorescent detection of muscle cells. The frontal cryosection was immunostained with a monoclonal antibody 12/101 **(J, K)** or an anti-PAX7 monoclonal antibody **(L, M)** to detect myofibers (white arrows in J, K) or myoblasts (white arrows in L, M) in the regenerated tail at day 5. Bar, 200 μm. A pair of black and white arrowheads marks the amputation plane.

**Figure 4:**
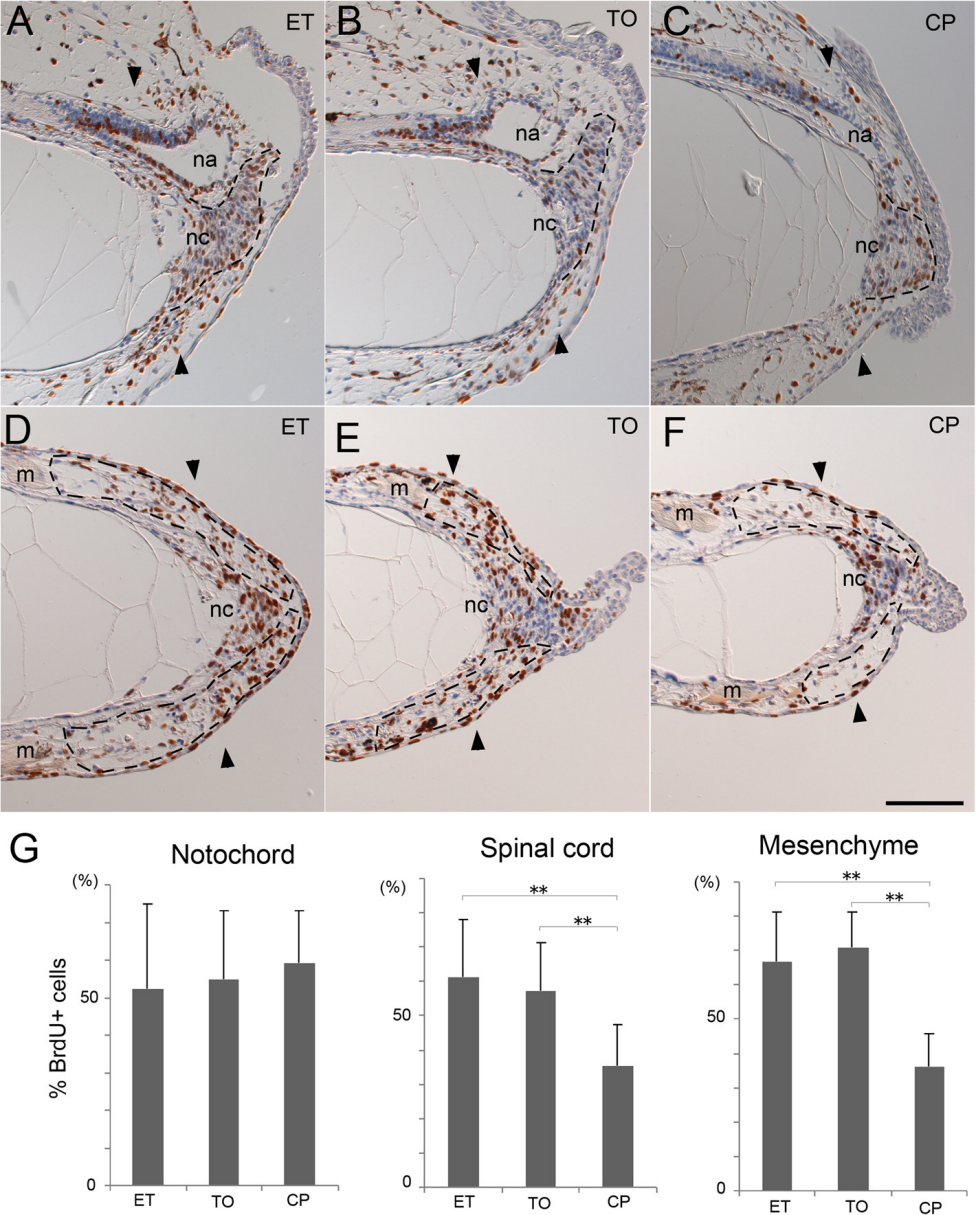
**Cyclopamine affects cell proliferation in the regenerating tail.** Tail-amputated tadpoles maintained in the presence of the indicated compound were incubated with BrdU for 12 h before fixation. **(A-F)** Immunohistochemical detection of proliferation cells at day 2.5. BrdU-incorporated cells (brown) were detected on sagittal **(A-C)** or frontal **(D-F)** section. Un-labeled nuclei were counter-stained with hematoxylin (blue). Dashed lines indicate the shapes of the regenerating notochords **(A-C)** and the mesenchymal regions **(D-F)** containing the myoblasts. nc, notochord. na, neural ampulla of the regenerating spinal cord. m, muscle. A pair of arrowheads marks the amputation plane. Bar, 100 μm. **(G)** Proliferation rate of cells in the regenerating tail. Mean rate of the BrdU-labeled cells was determined for the indicated tissue and shown with standard deviation. Detail is shown in Table 3. **, p-value <0.01.

In the normal tail regeneration process, undifferentiated notochord cells accumulate at the distal edge of the amputated notochord to make a mass of immature notochord cells at day 2 and proliferate extensively. The cells then align with a perpendicular orientation to the anterior to posterior axis and start to finally differentiate into large vacuole-containing cells after day 3 [5,23]. The cell mass of the notochord was formed in the cyclopamine-treated tail (Figures 3C, 4C, F), but the perpendicular alignment and vacuolation of the notochord cells was poorly accomplished in the cyclopamine-treated tails (Figure 2F, Figure 3C). The proliferation rate of the undifferentiated notochord cells was not affected by cyclopamine (Figure 4C, G, Table 3). These results together with the unaffected *bra3* expression (Figure 2K) show that cyclopamine does not affect the early stage of the notochord regeneration, but we concluded that hedgehog signaling is required for a later step of notochord differentiation, including cell alignment and vacuolation.

**Table 3:** Effect of Cyclopamine on proliferation of regenerating cells

**Tissues**	**Notochord**			**Spinal cord**			**Mesenchyme**		
**Chemicals**	**ET**	**TO**	**CP**	**ET**	**TO**	**CP**	**ET**	**TO**	**CP**
% BrdU^+^ cells	52.3	54.9	59.3	61.2	57.5	35.6	66.9	70.7	36.2
SD	22.5	18.2	13.8	16.7	13.6	11.6	14.5	10.5	9.6
number of sections	18	20	21	18	16	15	14	27	36
p-value (ET)	-	0.65	0.19	-	0.24	1.0 E-5	-	0.83	8.4 E-12
p-value (TO)	-	-	0.81	-	-	2.1 E-5	-	-	2.3 E-20

Tail-amputated st. 48 tadpoles were maintained in water containing ethanol (ET, 0.1%), tomatidine (TO, 2.5 μM) or cyclopamine (CP, 2.5 μM). Four tadpoles for each condition were fixed at day 2.5 after 12 h BrdU labeling. Mean rate of proliferating cells were determined on sagittal (notochord and spinal cord) or frontal (mesenchymal region) sections. Student’s *t-*test was used to identify differences between indicated groups.

### Cyclopamine affects spinal cord regeneration

The spinal cord grows posteriorly in close proximity to the regenerating notochord during tail regeneration. Immunostaining with an anti-NCAM antibody showed an impaired growth of the spinal cord in the cyclopamine-treated regenerated tail (Figure 3F). Expression of *sox2*, which is abundant in the regenerating spinal cord, is slightly reduced by the cyclopamine treatment (Figure 2K). A sagittal section showed that the neural ampulla of the regenerated spinal cord was small or not formed in the cyclopamine-treated tail (Figures 3C, 4C). Cyclopamine treatment significantly reduced the proliferation rate of cells in the neural ampulla region (Figure 4C, G, Table 3) but not in the proximal region of the spinal cord (data not shown).

### Cyclopamine inhibits muscle regeneration

Cyclopamine treatment greatly impaired the myofiber formation in the regenerated tail (Figure 3G-K). It suppressed up-regulation of *myoD* during tail regeneration (Figure 2K). It is known that Pax7-expressing myogenic cells migrate and proliferate to form new muscle tissue after tail amputation [5,24]. A few myogenic cells labeled with anti-Pax7 antibody were found in the regenerating tail treated with cyclopamine (Figure 3M), while many more Pax7 positive cells are in the control tadpole (Figure 3L). This is consistent with the result of RT-PCR showing that expression of *pax7* was down-regulated by cyclopamine treatment (Figure 2K). The proliferation rate of mesenchymal cells was reduced by cyclopamine in the region distal to the damaged muscle where Pax7-positive myogenic cells reside (Figure 4D-G, Table 3). We conclude that the hedgehog signal is required for muscle regeneration in the larval tail.

## Discussions

### Cyclopamine inhibits tail regeneration of *Xenopus* tadpole

In this study we showed that cyclopamine treatment resulted in a wide range of cellular defects including proliferation and differentiation during *Xenopus* tail regeneration. Muscle regeneration was especially inhibited. The fact that the number of Pax-7 positive myogenic cells and myofibers were significantly reduced is consistent with a previous report in which cyclopamine-treatment during axolotl tail regeneration results in a reduced number of Pax7-positive cells in regenerating blastema [13]. Our results suggest that the proliferation of the myogenic cells requires hedgehog signaling. The Pax-7 positive cells in the regenerating tail are derived from the satellite cells residing in the remaining muscle of the *Xenopus* tadpole tail [5,24,25]. As the promotive effect of Shh on proliferation and differentiation of the satellite cells has been reported in mouse and chicken [26,27], our results suggest that vertebrate muscle regeneration is regulated by a common mechanism involving hedgehog signaling.

We found a different effect of cyclopamine on the spinal cord regeneration between axolotl and *Xenopus*. Cyclopamine treatment does not affect proliferation of the regenerating spinal cord cells in axolotl tail regeneration [13]. On the other hand, it reduced the proliferation rate of cells in the neural ampulla region of the *Xenopus* spinal cord. Expression of *ptc-1* and *ptc-2* in the regenerating spinal cord (see Figure 1D, E) suggests a direct influence of hedgehog signal on spinal cord growth.

Cyclopamine treatment affected late steps of the notochord regeneration including the alignment of the immature cells and their terminal differentiation, although it did not affect the early step including the proliferation of the immature notochord cells. This result may be consistent with a previous report that *shh* is required for maintenance but not formation of the embryonic mouse notochord [28]. As the regenerating notochord cells express the hedgehog ligand, Shh, the hedgehog signal may regulate differentiation of notochord cells in an autocrine manner. Alternatively, Shh secreted by the notochord may regulate notochord regeneration indirectly by affecting other tissues, for example, the spinal cord that has been shown to be required for correct regeneration of the notochord [12]. The precise role of hedgehog signaling in the notochord cells will be determined using defined experimental systems, for example, culturing isolated notochord cells.

### Localized expression of *shh* causes the different tissue dependency in tail regeneration

Despite the differences in the affected tissues by cyclopamine treatment, this study together with previous reports show that hedgehog signaling is essential for the overall tail regeneration both in urodele and anuran [13]. The critical difference between *Xenopus* and axolotl is in the tissue-specific expression of *shh* in regenerating tail: *shh* is expressed exclusively in spinal cord in axolotl while it is expressed exclusively or predominantly in notochord in *Xenopu*s. Genes for other signaling molecules, including FGFs, Wnts and BMPs, are also required for tail regeneration in *Xenopus* and are expressed in a broader range of tissues in contrast to *shh*[7,12,23]. The different location of *shh* expressing cells is likely to be a main reason underlying the different tissue dependency for tail regeneration between urodele and anuran. *shh* is expressed in both the ventral spinal cord, namely the floor plate, and the notochord in vertebrate embryos. The original spinal cord in the *Xenopus* tadpole tail has a floor plate that expresses *shh*, while the regenerated one has no identifiable floor plate [23]. Therefore, the absence of *shh* expression in the regenerated spinal cord reflects the incomplete regeneration of the *Xenopus* spinal cord. Notochord, the main source of Shh in the regenerating *Xenopus* tadpole tail, is not formed in the regenerated tail of axolotl larva [13]. Regenerating tail in urodele larva forms cartilaginous tissue in place of the notochord, reflecting the normal developmental process in which notochord is replaced by cartilaginous vertebrae. On the other hand cartilage is never formed in the anuran tadpole tail. The difference in tissue-specific expression of *shh* between axolotl and *Xenopus* is, therefore, caused by the incomplete spinal cord regeneration in *Xenopus* and the absence of regenerated notochord in axolotl.

## Conclusion

In this study we clearly showed that overall regeneration of the *Xenopus* tadpole tail is inhibited by cyclopamine, a specific inhibitor for hedgehog signaling, consistent with a previous report using axolotl larva. We also showed that *shh* is exclusively or predominantly expressed in regenerating notochord but not in spinal cord in *Xenopus*, while it is expressed exclusively in spinal cord in axolotl. Our findings together with the previous report explain why the tissue dependency for tail regeneration is different between anuran and urodele larvae.

## Methods

### Surgery and animals

*Xenopus laevis* larvae were maintained at 22°C in 0.1% NaCl. The developmental stages were determined according to Nieuwkoop and Faber [29]. The stage 49 tadpoles were used for the tail amputation described previously [12]. The animals were handled in with a protocol approved by the animal care and use committee in University of Hyogo.

### Treatment with chemicals

Stock solutions (5 mM) of Cyclopamine (Enzo Life Science) and Tomatidine hydrochloride (Enzo Life Science) were prepared with ethanol and added into the feeding water at final concentration of 2.5 μM. Pumorphamine (Calbiochem) was dissolved in dimethyl sulfoxide at 10 mM and used at 0.25 μM. The concentrations used were determined empirically. Tadpoles were transferred into the water containing the chemicals immediately after amputation and the feeding water was replaced every day. Length of regenerated tail was measured from the center of the amputation plane to the distal epidermal tip. Length of regenerated notochord was measured using the different batch of tadpoles.

### Histological and immunological analyses

Histological analysis for staining with hematoxylin and eosin was performed as described previously [12]. Procedure for whole mount immunostaining was also described previously [23]. For frozen sections a tadpole were fixed in 7.4% formaldehyde, infiltrated in 18% sucrose, embedded in OCT compound and frozen at -80°C. Cryosection was made with cryostat (Leica CM3050S) and treated with a primary antibody and followed with Alexa Fluor 594-labeled secondary antibody (Molecular probes). Monoclonal antibodies 4d (anti-N-CAM) [30], 12/101 [31] and anti-PAX7 [32] were obtained from the Developmental Studies Hybridoma Bank at the University of Iowa (Iowa City, IA, USA).

Labeling of proliferating cells using bromodeoxyuridine (BrdU) was performed as described previously [12]. BrdU-labeled cells were detected with horseradish peroxidase-labeled secondary antibody and diaminobenzidine (DAB) coloring.

Apoptotic cells were detected by whole mount TUNEL staining using the In Situ Cell Death Detection Kit, POD (Roche Applied Science) according to manufacture’s protocol.

### Reverse transcription-polymerase chain reaction (RT-PCR)

The procedure for RNA extraction, cDNA synthesis and polymerase chain reaction was described previously [12]. The reaction conditions were empirically determined in each case within the linear range of amplification. Primers, annealing temperature and cycle number were as described in Additional file 2: Table S1.

### In situ hybridization

Procedures for whole mount and section in situ hybridizations were described previously [11,23]. Bluescript plasmids encoding *Xenopus laevis shh* (xl096o20), *patched-1* (xl008p21), *patched-2* (xl045b21), *gli-1* (xl088k18), *gli-2* (xl063k18) and *gli-3* (xl074e23) were characterized using the NIBB/NIG/NBRP Xenopus laevis EST database [33]. Complementary DNAs for *Xenopus bhh* was amplified from cDNA of the taildud embryo using primers (US: 5′-TGGAAGCTGGATTTGACTGGGTCTAC-3′; DS: 5′-GCTATACCAGTGAATGCCCATCTGCT-3′) and cloned into the PCR II-TOPO VECTOR (Life technologies) according to the manufacture’s protocol.

## Competing interests

The authors declare that they have no competing interests.

## Authors’ contributions

YT and MM performed the experiments. The paper was written by YT, KW and MM. The content is agreed by all the authors.

## Supplementary Material

Additional file 2: Table S1 Primer pairs and cycling conditions of the reverse transcription–polymerase chain reaction. Click here for file

Additional file 1: Figure S1 Apoptotic cells in the regenerating tail. Tail-amputated tadpoles maintained in the presence of 2.5 μM tomatidine (TO, A-F) or 2.5 μM cyclopamine (CP, D-F) were fixed at indicated days. Apoptotic cells were detected by the TUNEL staining (brown dots). Bar, 100 μm. Click here for file
