# Supplementary material for: Notochord-derived hedgehog is essential for tail regeneration in Xenopus tadpole
Source: BMC Dev Biol. 2014 Jun 18;14:27. doi: 10.1186/1471-213X-14-27 (PMC4074850; doi:10.1186/1471-213X-14-27)
Supplement: Additional file 2: Table S1 — Primer pairs and cycling conditions of the reverse transcription–polymerase chain reaction. [file 1471-213X-14-27-S2.doc]

Additional file 2: Table S1

Primer pairs and cycling conditions of the reverse transcription–polymerase chain reaction

| *Xenopus* |  | Anealing | Cycle |  |
| --- | --- | --- | --- | --- |
| genes | Sequences | temperature (ºC) | number | References |
| *shh* | U: 5'-CTTGGAGGAGTCGCTACATTATGAGGGGAG-3' | 55 | 32 | [12] |
|  | D: 5'-CGAACAGTGAATATGAGCTTTGGACTCGTA-3' |  |  |  |
| *bhh* | U: 5'-GAGAGGCACTGGCCACATTG-3' | 55 | 35 | [22] |
|  | D: 5'-ATCAGCCCCACCACATTTGA-3' |  |  |  |
| *c/dhh* | U: 5'-GAACAGCTATGGTTATGATG-3' | 55 | 35 | [22] |
|  | D: 5'-TGCACCTGAGTGCCATTCAC-3' |  |  |  |
| *ptc-1* | U: 5'-GGACAAGAATCGCAGAGCTG-3' | 60 | 30 | [34] |
|  | D: 5'-GGATGCTCAGGGAACCTTAC-3' |  |  |  |
| *ptc-2* | U: 5'-TTGTTCATTGGATTGCTGGTG-3' | 60 | 30 | [34] |
|  | D: 5'-CTCTTCCTGGTAGATATGCA-3' |  |  |  |
| *smo* | U: 5'-GCCCTGGGCACCACACATCAG-3' | 60 | 30 | This study |
|  | D: 5'-GATTAGCACAAAGCCAAAGGC-3' |  |  |  |
| *gli-1* | U: 5'-GAGCTAGTGACCCTGCAAG-3' | 60 | 30 | [34] |
|  | D: 5'-CATCGGGACCTGCTGTTTCC-3' |  |  |  |
| *gli-2* | U: 5'-CTCACAACATTGGCGGAGGAAAG-3' | 60 | 30 | This study |
|  | D: 5'-TCCGATTTTGGGCCAGGAATAG-3' |  |  |  |
| *gli-3* | U: 5'-CATTCCCAGCCATACCTGTTAGT-3' | 60 | 30 | This study |
|  | D: 5'-TGAGTTACGGGCTTGCTCATT-3' |  |  |  |
| *efi-a* | U: 5'-CAGGCCAGATTGGTGCTGGATATGC-3' | 60 | 28 | [35] |
|  | D: 5'-GCTCTCCACGCACATTGGCTTTCCT-3' |  |  |  |
| *myoD* | U: 5'-AACTGCTCCGATGGCATGATGGATTA-3' | 55 | 30 | [11] |
|  | D: 5'-GATGCTGGGAGAAGGGATGGTGATTA-3' |  |  |  |
| *pax7* | U: 5'-TACCGAGGCCCGTGTCCAGG-3' | 55 | 35 | This study |
|  | D: 5'-TGAGGTCCAACCCCACCGGG-3' |  |  |  |
| *sox-2* | U: 5'-TGATGTCAGTGCCCTGCAATATAACTCCAT-3' | 55 | 30 | [12] |
|  | D: 5'-GGAGCTGGATTCCGACTTGACTACCGAGCC-3' |  |  |  |
| *bra-3* | U: 5'-CACCCTCATTATAGGACAGAAACTTGTTTC-3' | 55 | 30 | [12] |
|  | D: 5'-GGATCTACTGGTGGACAAAACATTTTCTGT-3' |  |  |  |
| *wnt-5a* | U: 5'-GATCCTACAGCTCCTCCT-3' | 55 | 30 | [23] |
|  | D: 5'-CTAACGACCACCAGGAGCT-3' |  |  |  |
| *msx-1* | U: 5'-ATGGATCGCACTCCCCTACTGTAACTT-3' | 55 | 30 | [12] |
|  | D: 5'-TGCATCCTATTCAAGGGACGTTCTTC-3' |  |  |  |
| *ES1* | U: 5'-AGGTGGCAGTGATCCTTGCT-3' | 55 | 30 | [11] |
|  | D: 5'-TGCGTGCATCTGATCTATGT-3' |  |  |  |

34. Takabatake T, Takahashi TC, Takabatake Y, Yamada K, Ogawa M, Takeshima K: **Distinct expression of two types of Xenopus Patched genes during early embryogenesis and hindlimb development.** *Mech Dev* 2000*,* **98;** 99-104. Erratum in: *Mech Dev* 2001, **105:** 197.

35. Tazaki A, Kitayama A, Terasaka C, Watanabe K, Ueno N, Mochii M: **Macroarray-based analysis of tail regeneration in Xenopus laevis larvae.** *Dev Dyn* 2005, **233:** 1394-1404.
