# Supplementary figures and images for: Notochord-derived hedgehog is essential for tail regeneration in Xenopus tadpole
Source: BMC Dev Biol. 2014 Jun 18;14:27. doi: 10.1186/1471-213X-14-27 (PMC4074850; doi:10.1186/1471-213X-14-27)

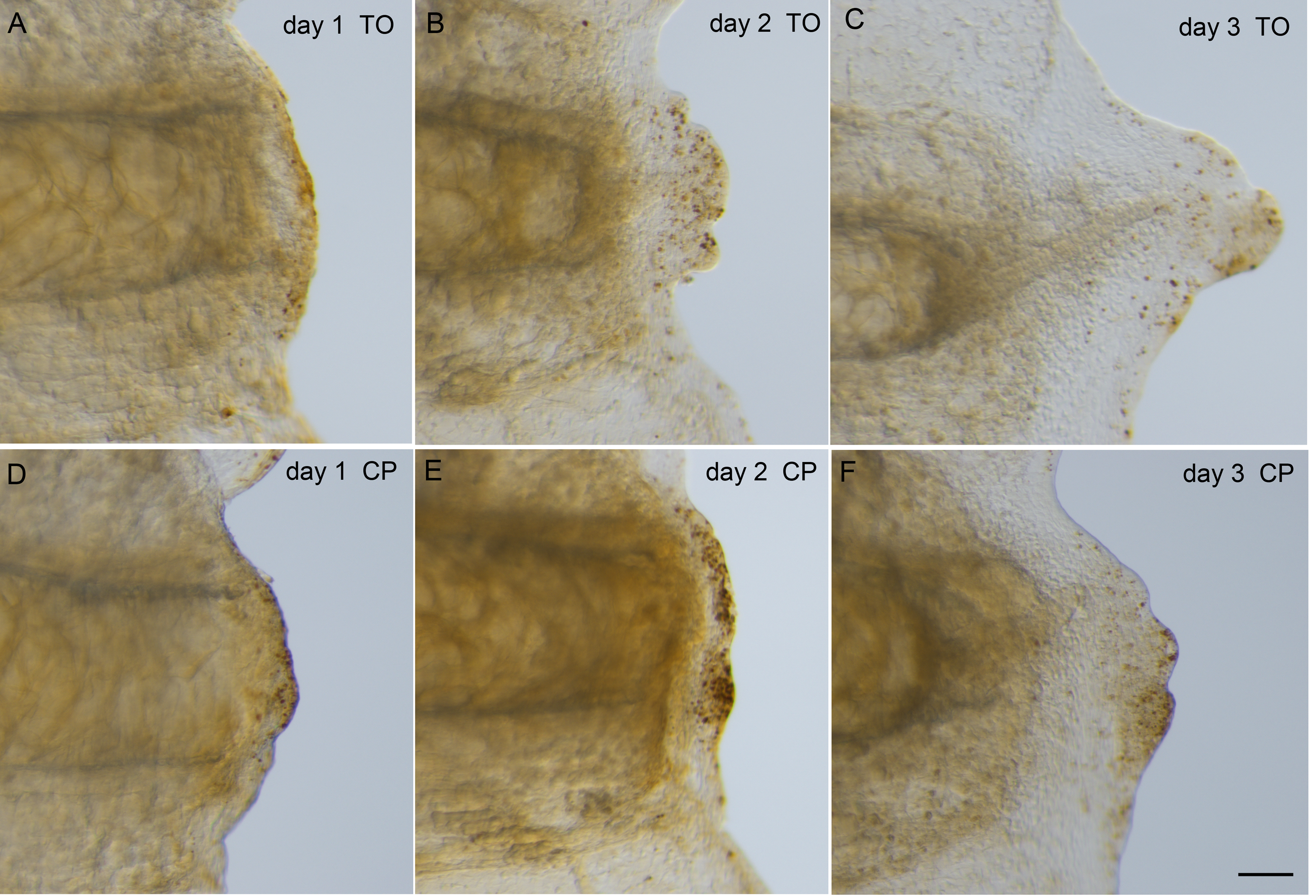

Supplement: Additional file 1: Figure S1 — Apoptotic cells in the regenerating tail. Tail-amputated tadpoles maintained in the presence of 2.5 μM tomatidine (TO, A-F) or 2.5 μM cyclopamine (CP, D-F) were fixed at indicated days. Apoptotic cells were detected by the TUNEL staining (brown dots). Bar, 100 μm. [file 1471-213X-14-27-S1.png]
